# Radiation dose reduction for CT lung cancer screening using ASIR and MBIR: a phantom study

**DOI:** 10.1120/jacmp.v15i2.4515

**Published:** 2014-03-06

**Authors:** Kelsey B. Mathieu, Hua Ai, Patricia S. Fox, Myrna Cobos Barco Godoy, Reginald F. Munden, Patricia M. de Groot, Tinsu Pan

**Affiliations:** ^1^ Department of Imaging Physics The University of Texas MD Anderson Cancer Center Houston TX USA; ^2^ Department of Biostatistics The University of Texas MD Anderson Cancer Center Houston TX USA; ^3^ Department of Diagnostic Radiology The University of Texas MD Anderson Cancer Center Houston TX USA

**Keywords:** computed tomography, ground‐glass opacity, iterative reconstruction

## Abstract

The purpose of this study was to reduce the radiation dosage associated with computed tomography (CT) lung cancer screening while maintaining overall diagnostic image quality and definition of ground‐glass opacities (GGOs). A lung screening phantom and a multipurpose chest phantom were used to quantitatively assess the performance of two iterative image reconstruction algorithms (adaptive statistical iterative reconstruction (ASIR) and model‐based iterative reconstruction (MBIR)) used in conjunction with reduced tube currents relative to a standard clinical lung cancer screening protocol (51 effective mAs (3.9 mGy) and filtered back‐projection (FBP) reconstruction). To further assess the algorithms' performances, qualitative image analysis was conducted (in the form of a reader study) using the multipurpose chest phantom, which was implanted with GGOs of two densities. Our quantitative image analysis indicated that tube current, and thus radiation dose, could be reduced by 40% or 80% from ASIR or MBIR, respectively, compared with conventional FBP, while maintaining similar image noise magnitude and contrast‐to‐noise ratio. The qualitative portion of our study, which assessed reader preference, yielded similar results, indicating that dose could be reduced by 60% (to 20 effective mAs (1.6 mGy)) with either ASIR or MBIR, while maintaining GGO definition. Additionally, the readers' preferences (as indicated by their ratings) regarding overall image quality were equal or better (for a given dose) when using ASIR or MBIR, compared with FBP. In conclusion, combining ASIR or MBIR with reduced tube current may allow for lower doses while maintaining overall diagnostic image quality, as well as GGO definition, during CT lung cancer screening.

PACS numbers: 87.57.Q‐, 87.57.nf

## I. INTRODUCTION

Lung cancer is the leading cause of death from cancer among men and women in the United States.^(1)^ According to the results of the National Lung Screening Trial (NLST), there was a 20% reduction in mortality from lung cancer among current or former heavy smokers who were screened with low‐dose computed tomography (CT) compared with chest radiography.^(2)^ Although this encouraging news is increasing interest in using CT for lung cancer screening, concerns about radiation dosage from medical imaging are also being raised; this is particularly true when considering extension of screening to include patients who have never smoked. While these concerns have led to efforts to decrease radiation dosage during CT imaging, it is critical that diagnostic image quality is not sacrificed in favor of dose reduction. In particular, it is extremely important that reductions in dose during CT lung screening examinations do not limit the detectability of ground‐glass opacities (GGO), which are often indicative of premalignant lesions or early adenocarcinomas.^(3)^

Our hypothesis was that advanced iterative image reconstruction techniques would improve nodule definition and overall image quality (for a given dose), which in turn would allow for reduction of the radiation dose typically associated with CT lung cancer screening. Thus, the purpose of this study was to investigate the feasibility of using the image reconstruction techniques of adaptive statistical iterative reconstruction (ASIR) and model‐based iterative reconstruction (MBIR) in conjunction with lowering tube current to minimize radiation doses, while maintaining GGO definition and overall image quality.

## II. MATERIALS AND METHODS

### A. Phantoms

**Figure 1:**
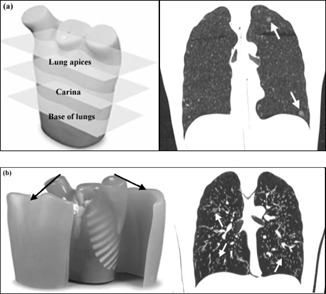
Two anthropomorphic phantoms were used as part of the quantitative assessment performed in this study, including (a) a lung screening phantom, and (b) a multipurpose chest phantom; fat slabs were added to the multipurpose chest phantom to better reflect an average‐sized patient. Coronal CT views are shown alongside the photographs to illustrate the longitudinal location of several of the low‐contrast targets and simulated GGOs within each phantom. (Images reprinted with permission from Kyoto Kagaku).

A lung screening phantom (LSCT001; Kyoto Kagaku, Kyoto, Japan) and a multipurpose chest phantom with added fat slabs (N1 “LUNGMAN”; Kyoto Kagaku) were used in this study and are shown in Fig. 1. The lung screening phantom contained built‐in, low‐contrast targets (urethane resin), which were intended to represent GGOs, at the level of the lung apices, the carina, and the base of the lungs. The targets in the left and right lungs (sizes: 2, 4, 6, 8, and 10 mm and 4, 6, 8, 10, and 12 mm, respectively) had contrasts of 270 and 100 Hounsfield units (HU), respectively, relative to the background (polysterol foam). The lungs of the multipurpose chest phantom (polyurethane) were implanted with 12 simulated lung nodules of four sizes (5, 8, 10, and 12 mm) and three Hounsfield units (‐800 HU (low attenuation GGOs; urethane foam), −630 HU (high attenuation GGOs; urethane foam), and 100 HU (solid nodules; polyurethane, SZ50, and hydroxyaptite)). The added fat slabs gave the multipurpose chest phantom a diameter measuring (from left‐to‐right) approximately 41 cm, which was 6 cm larger than the diameter of the lung screening phantom. While both phantoms were well‐suited for the quantitative analysis, the multipurpose chest phantom was better suited for our qualitative assessment, as it was more realistic for modeling patient size and anatomy.

### B. Data acquisition and image reconstruction

The phantoms were scanned on a Discovery CT750 HD scanner (GE Healthcare, Waukesha, WI) using a clinical lung cancer screening protocol adapted from the NLST^(4)^ (${\text{CTDI}}_{\text{vol}}=3.9\;\text{mGy}$; 51 effective mAs (125 mA, 0.4 s rotation time, 0.984 pitch), 120 kVp, standard kernel, 40 mm beam collimation, and 2.5 mm reconstructed slice width with a 1.25 mm reconstructed interval). The scans were also repeated using lower tube currents (100, 75, 50, 25, and 10 mA); fixed tube currents were used to maintain consistency with both the NLST technique and the clinical lung screening protocol used at our facility at the time of this study. All images were reconstructed with filtered back‐projection (FBP), 50% ASIR (ASiR; GE Healthcare), and MBIR (Veo; GE Healthcare); a standard reconstruction kernel was used for FBP and 50% ASIR (a blend of 50% ASIR was chosen based on prior literature^(5)^).

### C. Quantitative analysis

Image noise (measured as the image standard deviation) was compared across scan techniques by drawing regions of interest (ROIs) in the six largest low‐contrast targets in the lung screening phantom, which were located at the level of the lung apices, the carina, and the base in both the left and right side of the lungs. ROIs were also drawn in the background of the lungs to assess the contrast‐to‐noise ratio (CNR) using the method described by Funama et al.^(6)^ Furthermore, ROIs were drawn in the background of the lungs and in the eight simulated GGOs in the multipurpose chest phantom to assess nodule noise and CNR.

### D. Qualitative analysis

#### D.1 Nodule definition

Scan technique and reconstruction identifiers were removed from the CT images of the multipurpose chest phantom and three board‐certified thoracic radiologists with 3, 7, and 22 years of experience reviewed the images on a PACS workstation (iSite; Philips Healthcare, Cleveland, OH) in a dark reading room under normal clinical reading conditions. The readers were shown single images of each of the 12 implanted nodules. The readers reviewed 54 randomly‐ordered images per nodule; the 54 images consisted of 18 combinations of effective mAs and reconstruction technique (six effective mAs and three reconstruction techniques) with three replicates per combination. All 54 images of each nodule were placed within a single series and each series, which only contained images of the specified nodule, was presented separately to readers. On a reference image, which was acquired and reconstructed using the clinical protocol (51 effective mAs, FBP), readers were shown the location of each nodule; the reference image for each nodule was viewed side‐by‐side against each of the 54 images of the nodule. Readers were blinded to the fact that three copies of the reference image were intermixed with the 54 images and that they were shown three replicates for each combination of effective mAs and reconstruction technique. Readers were instructed to rate their ability to visualize each nodule for all 54 images compared with the reference image using a scale from 0 to 5 (0 = nodule cannot be seen, 1 = nodule is much less defined than reference, 2 = nodule is slightly less defined than reference, 3 = nodule definition is same as reference, 4 = nodule is slightly more defined than reference, 5 = nodule is much more defined than reference). For each of the 12 series (nodules), all ratings were assigned in a single sitting. Data were pooled across the readers and replicates by nodule density (e.g., solid nodule) and nodule size (e.g., < 8 mm) to calculate the mean rating for each combination of effective mAs and reconstruction algorithm.

#### D.2 Overall image quality

Readers were also shown a series of images, which covered the entire lungs and thus contained all 12 implanted lung nodules, for each combination of effective mAs and reconstruction technique. The readers were instructed to rate and record their impression of the overall diagnostic image quality of the 54 randomly‐ordered series of images (18 combinations of effective mAs and reconstruction technique; three replicates per combination) relative to a reference series, which was acquired and reconstructed using the clinical protocol (51 effective mAs, FBP). Ratings were assigned in one sitting on a scale of 1‐5 (1 = overall diagnostic image quality is much worse than reference, 2 = overall diagnostic image quality is slightly worse than reference, 3 = overall diagnostic image quality is same as reference, 4 = overall diagnostic image quality is slightly better than reference, 5 = overall diagnostic image quality is much better than reference). Ratings were pooled across the three readers and three replicates to calculate the average rating of overall image quality for each effective mAs and reconstruction technique.

### E. Statistical analysis

As part of the quantitative analysis performed on both anthropomorphic phantoms, a generalized linear mixed model (GLMM) for continuous outcomes was used to assess the CNR and image noise as a function of effective mAs and image reconstruction technique. For the lung screening phantom, lung location (apices, base, carina) was also included in the model. In order to account for comparing each effective mAs and reconstruction technique to the clinical protocol (51 effective mAs, FBP), Dunnett's method was used to adjust the p‐values.^(7)^

For the qualitative portion of the study, a separate GLMM was used to assess whether each effective mAs and reconstruction technique produced a rating less than “3” given that a rating equal to “3” represented no change from the reference (i.e., the clinical protocol). The model allowed a random reader effect and accounted for repeated nodules in the ratings of nodule definition. To account for comparing each of the 18 different effective mAs and reconstruction technique combinations against a rating of “3,” the Holm‐Bonferroni method was used with a family wise error rate of 5%;^(8)^ no adjustments for multiplicity were made to account for nodule density or size. Cohen's kappa statistic was used to assess both the intrareader and interreader agreement.^(9)^ All statistical analyses were performed using SAS 9.3 for Windows (SAS Institute Inc., Cary, NC).

## III. RESULTS

### A. Quantitative analysis

In the lung screening phantom, we observed a significant overall effect for reconstruction technique and effective mAs (p=0.007) and some evidence of an effect for lung location (after taking into account reconstruction and effective mAs) (p=0.065). In general, FBP produced higher noise estimates, followed by ASIR, and then MBIR; this relationship was most noticeable at 4 and 10 effective mAs, particularly in the apices. When considering each specific combination of reconstruction technique and effective mAs, using FBP at 4 effective mAs produced significantly higher mean noise compared to the clinical protocol (51 effective mAs, FBP); however, no significant differences (relative to the clinical protocol) were observed for other combinations of reconstruction and effective mAs. For the CNR, no statistically significant differences were observed, and the model showed no significant effects for the combination of reconstruction technique and effective mAs or for lung location. However, when comparing the mean CNR and image noise across reconstructions, our results indicated that dose could be reduced by 40% (to 30 effective mAs) when using ASIR or 80% (to 10 effective mAs) when using MBIR, compared with the clinical protocol (51 effective mAs, FBP), while maintaining a similar level of mean noise and CNR; Table 1 details these result alongside the corresponding CT images.

**Table 1:**
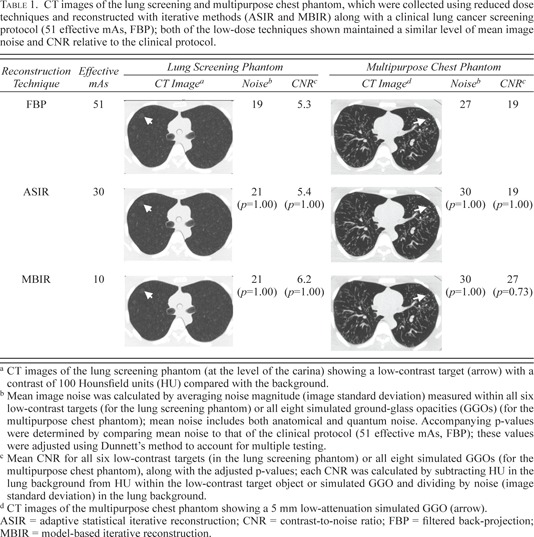
CT images of the lung screening and multipurpose chest phantom, which were collected using reduced dose techniques and reconstructed with iterative methods (ASIR and MBIR) along with a clinical lung cancer screening protocol (51 effective mAs, FBP); both of the low‐dose techniques shown maintained a similar level of mean image noise and CNR relative to the clinical protocol

In the multipurpose chest phantom, no combination of reconstruction technique and effective mAs produced significantly lower or higher noise or CNR compared with the clinical protocol (51 effective mAs, FBP). When considering the mean CNRs, we found that, in images acquired at 30 effective mAs (40% dose reduction) when using ASIR or 10 effective mAs (80% dose reduction) when using MBIR, the average CNR (across all eight GGOs) was equal or greater than in images acquired at 51 effective mAs and reconstructed using FBP Similar mean image noise (within all GGOs) in these low‐dose images reconstructed with ASIR and MBIR compared with the clinical protocol (51 effective mAs, FBP) was also observed. These results, along with the corresponding CT images for the aforementioned combinations of reconstruction technique and effective mAs, are presented in Table 1.

### B. Qualitative analysis

Table 2 lists the readers' mean ratings of both nodule definition and overall diagnostic image quality in the multipurpose chest phantom; statistical significance of these ratings (after accounting for multiple comparisons) is also indicated. As Table 2 shows, at 4 effective mAs, all three reconstruction techniques had mean ratings significantly less than “3” for both nodule definition (regardless of nodule density or size) and overall diagnostic image quality. Despite the significantly lower mean ratings of nodule definition at 4 effective mAs, the fact that readers never assigned a rating of “0” indicates that (even at very low doses) they were able to visualize all nodules. At 20, 30, and 41 effective mAs, no mean reader ratings of nodule definition or overall image quality were significantly less than “3” for MBIR, ASIR, and FBP, respectively. On the other hand, for 41 and 51 effective mAs, MBIR produced mean overall image quality and nodule definition (across solid nodules, large nodules, all GGOs, and all nodules) ratings significantly higher than “3.”

**Table 2:** Mean ratings^a^ for nodule definition and overall diagnostic image quality as evaluated using the multipurpose chest phantom

*Effective mAs* ^b^	${\text{CTDI}}_{\text{vol}}\;(\text{mGy})$ ^c^	*Reconstruction Technique*	*Solid Nodules*	*High Attenuation GGOs*	*Low Attenuation GGOs*	*All GGOs*	*Small Nodules (5& 8 mm)*	*Large Nodules (10 & 12 mm)*	*All Nodules*	*Overall Diagnostic Image Quality*
4	0.3	FBP	**2.5**	**1.5**	**1.2**	**1.3**	**1.8**	**1.7**	**1.7**	**1.1**
(92%)		ASIR	**2.2**	**1.4**	**1.3**	**1.4**	**1.7**	**1.6**	**1.7**	**1.1**
		MBIR	**2.2**	**1.5**	**1.7**	**1.6**	**1.7**	**1.9**	**1.8**	**1.3**
10	0.8	FBP	**2.8**	**2.3**	**2.0**	**2.1**	**2.3**	**2.4**	**2.4**	**2.0**
(80%)		ASIR	**2.8**	**2.3**	**2.2**	**2.2**	**2.4**	**2.5**	**2.4**	2.4
		MBIR	3.0	2.8	**2.4**	**2.6**	**2.7**	**2.7**	**2.7**	**2.0**
20	1.6	FBP	**2.8**	2.8	**2.4**	**2.6**	**2.6**	**2.7**	**2.6**	2.6
(60%)		ASIR	3.0	2.9	**2.6**	2.8	2.9	2.8	2.9	2.7
		MBIR	3.1	3.0	2.9	3.0	3.0	3.0	3.0	2.7
30	2.4	FBP	3.0	3.0	**2.7**	2.8	2.8	2.9	2.9	3
(40%)		ASIR	3.1	3.0	2.8	2.9	3.0	3.0	3.0	3.1
		MBIR	3.1	3.3	3.2	3.2	3.2	3.2	3.2	3.2
41	3.1	FBP	3.0	3.0	3.0	3.0	3.0	3.0	3.0	3
(20%)		ASIR	3.1	3.2	3.1	3.1	3.1	3.1	3.1	3.0
		MBIR	**3.2**	3.3	3.2	**3.3**	3.2	**3.3**	**3.2**	3.4
51	3.9	FBP	2.9	3.0	3	3.0	3.0	3.0	3.0	3.1
		ASIR	3.1	3.2	3.0	3.1	3.1	3.1	3.1	3.3
		MBIR	**3.3**	3.3	3.2	**3.3**	3.2	**3.4**	**3.3**	**3.8**

^a^ Reader ratings are relative to a reference image or series, which was acquired using the clinical lung cancer screening protocol (51 effective mAs, FBP). Data were pooled across three readers and three replicates to calculate the mean ratings. Mean ratings significantly less than “3” (after accounting for multiple testing) appear in bold‐faced font; ratings significantly greater than “3” are also bold‐faced.

^b^ Dose reduction percentages (relative to the clinical protocol (51 effective mAs, FBP)) are given below the effective mAs.

^c^ ${\text{CTDI}}_{\text{vol}}$ estimates were obtained from the scanner console and are relative to a 32 cm CTDI phantom.

ASIR=adaptive statistical iterative reconstruction; CTDI=computed tomography dose index; FBP=filtered back‐projection; GGOs=ground‐glass opacities; MBIR=model‐based iterative reconstruction.

**Figure 2:**
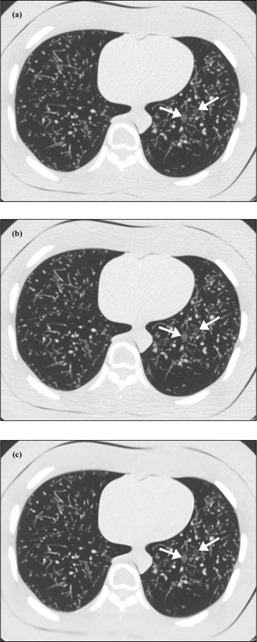
CT images of the multipurpose chest phantom showing the 8 mm low‐attenuation (anterior) and high‐attenuation (posterior) simulated ground‐glass opacities (GGOs). These images were acquired using (a) a clinical lung cancer screening protocol (51 effective mAs) and reconstructed with filtered back‐projection (FBP), or (b) and (c) a reduced tube current protocol (20 effective mAs) and reconstructed with adaptive statistical iterative reconstruction (ASIR) or model‐based iterative reconstruction (MBIR), respectively.

When comparing the mean reader ratings of nodule definition, the ratings for solid nodules were generally higher than for GGOs (for a given mAs and reconstruction technique). Furthermore, the mean reader ratings for the high attenuation GGOs were generally higher than for the low attenuation GGOs. With respect to nodule size, the ratings for nodules larger than 8 mm were generally the same or higher than those for nodules 8 mm or smaller. Across all eight GGOs and all 12 nodules, the mean reader ratings when using 20 effective mAs (60% dose reduction relative to the clinical protocol) and ASIR or MBIR were not observed to be significantly less than a mean rating of “3.” However, in general, the readers had higher mean rating of nodule definition for images reconstructed with MBIR than with ASIR. Figure 2 shows axial views of the phantom scanned at the aforementioned low‐dose protocols alongside the clinical protocol.

Table 2 also shows that the mean reader ratings of overall diagnostic image quality (for a given dose) when using either ASIR or MBIR were equal to or higher than when using FBP. For a dose reduction of 60% (i.e., 20 effective mAs), the mean ratings of diagnostic image quality were not significantly lower than “3” when using either ASIR or MBIR. While the mean rating for 10 effective mAs and ASIR was determined not to be statistically significant and the rating for MBIR (at 10 effective mAs) was significant, in general, the mean reader ratings for image quality when using MBIR were higher than when using ASIR (e.g., at 30 effective mAs, the average rating was 3.2 when using MBIR versus 3.1 with ASIR).

Despite the fact that the clinical protocol (51 effective mAs, FBP) was used as the reference, reader ratings for this technique were not always equal to “3”. Three out of 108 reader ratings of nodule definition given for the clinical protocol were different than “3;” for overall image quality, one out of nine ratings of the clinical protocol differed from “3.” All four ratings differing from “3” were given by the reader with the least clinical experience. Regarding interreader agreement, the kappa statistics (across all readers and replicates) were 0.31 and 0.34 for overall image quality and nodule definition (across all 12 nodules), respectively; these kappa statistics indicate fair interreader agreement using the Landis interpretation criteria.^(10)^ Interreader agreement for nodule definition was higher when pooling the data reconstructed using FBP versus ASIR versus MBIR (kappa statistics: 0.40 versus 0.38 versus 0.22, respectively). The results for intrareader agreement were that both reader 1 and reader 3 had moderate agreement for overall image quality (kappa statistics: 0.47 and 0.50, respectively) and nodule definition (across all 12 nodules) (kappa statistics: 0.60 and 0.51, respectively), while reader 2 had substantial intrareader agreement (kappa statistic: 0.72 for overall image quality and 0.76 for nodule definition).

## IV. DISCUSSION

In this study, we showed that, by lowering tube current and using ASIR or MBIR to reconstruct the CT images, radiation dose can potentially be reduced by at least 60% (to 20 effective mAs (1.6 mGy)) compared to a clinical lung cancer screening protocol (51 effective mAs (3.9 mGy), FBP), while achieving roughly the same mean reader ratings of overall diagnostic image quality and GGO definition. This conclusion was reached based on qualitative image analysis of a multipurpose chest phantom. However, the potential for even greater dose reduction when using MBIR was observed through quantitative analysis of the multipurpose chest phantom, as well as a lung screening phantom (80% dose reduction potential). The difference in dose reduction potential as determined by our qualitative versus quantitative assessment may be explained by the fact that: (1) traditional metrics for objectively judging image quality (i.e., image noise magnitude and CNR) are based on linear reconstruction methods and may not be appropriate given the nonlinear behavior of iterative reconstruction techniques;^11^, ^12^ and (2) iterative reconstruction methods can affect the noise texture of the CT images and, because this is not taken into account through traditional metrics, such metrics may not be able to accurately predict diagnostic impact. Therefore, even in the case of an iteratively reconstructed image with a lower noise magnitude compared to FBP, changes to the noise texture can impair an observer's ability to discern low‐contrast objects. For this reason, using quantitative metrics alone is not adequate for evaluating low‐contrast detectability or overall image quality, and reader studies remain the gold standard for determining appropriate dose levels prior to implementation of new iterative reconstruction techniques into clinical practice. Another explanation for the difference in our quantitative‐versus qualitative‐based conclusions is that the conditions under which dose reduction potential was derived differed due to the limited power of the statistical analysis performed in the quantitative portion of the study. In the quantitative portion, the data were too variable to be statistically significant, which was likely due to the fact that a much smaller number of data points were analyzed compared to that of the qualitative study; for this reason, mean CNR and noise were instead used to quantify dose reduction. In the case of the reader study, many more data points were included, which explains why the significance results were more consistent with the reader ratings.

An important consideration when implementing iterative reconstruction techniques is reader performance. The lower interreader agreement we observed for MBIR (kappa statistic: 0.22) relative to FBP (kappa statistic: 0.40) may be a limitation of MBIR. However, as radiologists become more accustomed to the unfamiliar texture of MBIR images, which our readers considered to be slightly blurry and artificially oversmoothed (particularly at very low doses), interreader agreement may improve. Despite the readers' relatively low agreement, the unfamiliar appearance of the MBIR images did not affect their ability to visualize the nodules. In fact, several of the mean ratings for MBIR were significantly higher than “3” (the rating assigned when overall diagnostic image quality or nodule definition was equal to that of the clinical protocol (51 effective mAs, FBP)), indicating that (in some cases) our readers may have preferred MBIR to FBP. However, the fact that readers in this study did not assign a rating of “5” to any of the MBIR images suggests that they considered image quality and nodule definition to be only slightly improved by MBIR. While kernels cannot be used with MBIR, the readers believed that MBIR could be enhanced in the future by offering a postprocessing option that would sharpen the edges in the MBIR images (similar to the lung kernel used with FBP).

For institutions that do not have access to MBIR (or are limited by its lengthy reconstruction times), ASIR may offer an improvement over FBP, given that the readers in this study generally rated nodule definition and overall diagnostic image quality higher when using ASIR compared with FBP (for a given dose). For institutions without access to either ASIR or MBIR, our results showed that, when using FBP, the clinical dose could be lowered to 41 effective mAs (3.1 mGy), while achieving similar mean reader ratings for nodule definition and overall image quality.

The strength of this study compared to other studies evaluating the use of ASIR and MBIR in conjunction with reduced dose is that it represents a more thorough qualitative and quantitative analysis (including the use of two phantoms) for the specific application of lung cancer screening with a focus on GGO definition.^13^, ^14^, ^15^ Despite the study strengths, there were several limitations. One such limitation was that a standard kernel rather than a lung kernel, which the readers may have preferred, was used for FBP and ASIR in order to maintain consistency with NLST specifications and the clinical protocol used at our facility. Also, for the same reason, fixed tube currents were used; however, tube current modulation should be considered in the future to promote further dose reduction.

A limitation specific to the reader portion of the study was that nodule locations were fixed. Because image quality could differ throughout the lungs (our quantitative analysis of the lung screening phantom did provide some evidence of an effect for lung location (p=0.0647)), nodule definition may have been rated differently if the same nodule was in a different location within the lungs. However, because nodule definition was rated relative to a reference acquired at the same anatomic location, we can assume that this did not have a large impact on our conclusions.

The nodules and low‐contrast targets implanted in both anthropomorphic phantoms used in this study were perfectly spherical or cylindrical, which is unlikely to be the case in patients, and were also of uniform density, and thus did not accurately model the GGOs seen in humans. Furthermore, we only assessed pure GGOs in this study and did not consider part‐solid GGOs, which have a higher incidence of malignancy than either pure GGOs or solid nodules.^(16)^ Although we did not examine part‐solid GGOs, pure GGOs tend to have an intrinsically lower attenuation and, therefore, lower conspicuity, making them more difficult to detect than GGOs with a solid component; thus, we assume that our analysis of pure GGOs would also apply to part‐solid GGOs.

Another limitation of our study was that we performed an evaluation rather than a detection reader study. Readers were aware of the nodule locations and were not shown images without nodules; thus, we did not determine the influence of the different effective mAs and reconstruction technique combinations on the readers' accuracy for detection of nodules. While no ratings of “0” were given, it is possible that the readers would not have detected all of the nodules if they had not been shown their locations on a reference image. Additionally, because our study only evaluated reader preference, even statistically significant differences in mean reader ratings do not necessarily translate to a loss of nodule detectability or diagnostic image quality. Furthermore, slightly improved or degraded visualization of lung nodules (i.e., ratings of “2” or “4” versus “3”) may not reflect a meaningful difference to experienced radiologists and reader preferences may vary across institutions. That being said, this study does not allow for determination of the dose cutoff that translates to a loss in nodule detectability or image quality, and it is possible that even lower doses than what we reported could potentially be used while still accomplishing the clinical imaging task. Therefore, further studies are needed to evaluate radiologists' abilities to detect and characterize subsolid pulmonary nodules in patient datasets at lower doses and with new iterative reconstruction algorithms.

## V. CONCLUSION

Combining the advanced reconstruction techniques of ASIR or MBIR with reduced tube currents may allow for much lower radiation doses while maintaining overall image quality, as well as GGO definition, during CT lung cancer screening.

## ACKNOWLEDGMENTS

The authors would like to thank Dr. Adam Chandler for his assistance in obtaining the MBIR reconstructions and Dr. Jiang Hsieh for answering technical questions related to MBIR. We would also like to thank Dr. Brian Hobbs for participating in our initial discussions of the study design and Roland Bassett and Auston Wei for advice regarding the statistical analysis. Finally, we would like to acknowledge our funding sources: Cancer Center Support Grant (NCI Grant P30 CA016672) and a research agreement between The University of Texas MD Anderson Cancer Center and GE Healthcare.
